# Distinct Pro-Inflammatory Mechanisms Elicited by Short and Long Amosite Asbestos Fibers in Macrophages

**DOI:** 10.3390/ijms242015145

**Published:** 2023-10-13

**Authors:** Riccardo Leinardi, Jasmine Rita Petriglieri, Amandine Pochet, Yousof Yakoub, Marie Lelong, Alain Lescoat, Francesco Turci, Valérie Lecureur, François Huaux

**Affiliations:** 1 Louvain Centre for Toxicology and Applied Pharmacology (LTAP), Institute de Recherche Expérimentale et Clinique (IREC), Université Catholique de Louvain (UCLouvain), 1200 Brussels, Belgium; amandine.pochet@uclouvain.be (A.P.); yousof.yakoub@uclouvain.be (Y.Y.); francois.huaux@uclouvain.be (F.H.); 2 “G. Scansetti” Interdepartmental Centre for Studies on Asbestos and Other Toxic Particulates, University of Turin, 10125 Turin, Italy; jasminerita.petriglieri@unito.it (J.R.P.); francesco.turci@unito.it (F.T.); 3 Department of Earth Sciences, University of Turin, 10125 Turin, Italy; 4 Université de Rennes, CHU Rennes, INSERM, EHESP, Irset (Institut de Recherche en Santé, Environnement et Travail)—UMR_S 1085, 35000 Rennes, France; marie.lelong@univ-rennes.fr (M.L.); alain.lescoat@chu-rennes.fr (A.L.); valerie.lecureur@univ-rennes.fr (V.L.); 5 Department of Internal Medicine and Clinical Immunology, Rennes University Hospital, 35000 Rennes, France; 6 Department of Chemistry, University of Turin, 10125 Turin, Italy

**Keywords:** amosite asbestos, inflammation, immunogenic cell death, pyroptosis, apoptosis, gasdermin D, TLR4, IL-1α, macrophages, TNF-α

## Abstract

While exposure to long amphibolic asbestos fibers (L > 10 µm) results in the development of severe diseases including inflammation, fibrosis, and mesothelioma, the pathogenic activity associated with short fibers (L < 5 µm) is less clear. By exposing murine macrophages to short (SFA) or long (LFA) fibers of amosite asbestos different in size and surface chemistry, we observed that SFA internalization resulted in pyroptotic-related immunogenic cell death (ICD) characterized by the release of the pro-inflammatory damage signal (DAMP) IL-1α after inflammasome activation and gasdermin D (GSDMD)-pore formation. In contrast, macrophage responses to non-internalizable LFA were associated with tumor necrosis factor alpha (TNF-α) release, caspase-3 and -7 activation, and apoptosis. SFA effects exclusively resulted from Toll-like receptor 4 (TLR4), a pattern-recognition receptor (PRR) recognized for its ability to sense particles, while the response to LFA was elicited by a multifactorial ignition system involving the macrophage receptor with collagenous structure (SR-A6 or MARCO), reactive oxygen species (ROS) cascade, and TLR4. Our findings indicate that asbestos fiber size and surface features play major roles in modulating ICD and inflammatory pathways. They also suggest that SFA are biologically reactive in vitro and, therefore, their inflammatory and toxic effects in vivo should not be underestimated.

## 1. Introduction

Asbestos is the commercial name by which six mineral fibers, five amphiboles (including actinolite asbestos, amosite (fibrous grunerite), anthophyllite, crocidolite, and tremolite asbestos, and one serpentine (chrysotile) are commonly known [1,2,3,4]. Exposure to asbestos fibers is associated with an increased risk of developing long-latency diseases such as pulmonary fibrosis, lung cancer, and pleural and peritoneal mesothelioma [5,6]. The mechanisms by which asbestos causes diseases are not fully understood [7,8]. While fibers longer than 10 µm are associated with an increased risk of asbestos-related diseases, fibers shorter than 5 µm are considered less pathogenic [9,10]. This cut-off was historically proposed as a dimensional limit to differentiate short from long fibers [11]. It is reported that alveolar macrophages (AM) are in charge of removing asbestos fibers deposited in the alveoli following inhalation [12]. Short fibers are fully engulfed (internalized) by macrophages and removed by lung clearance more efficiently than long ones, which tend to persist in the lungs [10,11,13]. This process results in frustrated phagocytosis and macrophage death, leading to the long-term retention of the fibers in the tissue, accompanied by the establishment of a pro-inflammatory (via NLRP3 inflammasome activation) and mutagenic microenvironment [7]. Also, the interaction of asbestos fibers with macrophages results in the production of intracellular reactive oxygen species (ROS), contributing to DNA damage, genomic instability, and, in the context of mesothelioma, malignant transformation of mesothelial cells [14,15]. Available data support the conclusion that the toxicity of asbestos relies on fiber dimensions [9]. However, the possibility that short fibers may induce detrimental effects cannot be fully discarded [11,16,17]. 

The release of pro-inflammatory cytokines by AM, including IL-1β and TNF-α, in vivo and in vitro during asbestos exposure is well described [18,19]. However, a mechanistic model relating macrophage activation and response to the specific dimension of the fibers is still missing. Macrophages express a large variety of cell-surface receptors (also called “pattern recognition receptors” or PRRs) [20]. Their main role is to recognize and internalize pathogens (pathogen-associated molecular patterns, PAMPs), pathogenic particles, apoptotic cells, and cell debris [20]. Scavenger receptors (SR) are transmembrane glycoproteins found on the surface of several cell types, including macrophages. They are divided into different classes based on structure and function [21]. SR class A, which includes macrophage receptors with collagenous structures (SR-A6 or MARCO), SR-A1, and CD204 (SCARA1) subtypes, was found to play a role in the internalization of unopsonized inorganic particles by AM, such as titanium dioxide (TiO_2_), silica (SiO_2_), and diesel exhaust particles [22]. Furthermore, it was reported that MARCO-deficient mice showed less fibrosis following exposure to chrysotile fibers, suggesting their possible involvement in the response to asbestos [23]. Additional PRRs are toll-like receptors (TLRs) and type-I transmembrane glycoproteins sensing infectious agents via PAMP recognition [24,25]. TLRs recognize their ligands either on the surface of cells (TLR1-6 and TLR-10) or intracellularly (TLR3, 7, 8, 9, 11, 12, and 13) [24]. TLR2 and TLR4 are involved in the pro-inflammatory response to environmental inorganic particulate matter (PM) by activating the production and release of several cytokines, including IL-6, IL-8, and TNF-α, in vitro [26]. TLR4 stimulation with LPS induces pro-IL-1α upregulation [27]. Also, TLR4 and its downstream inflammatory cascade were found to modulate silica and carbon nanotube-induced inflammation [28,29]. Whether TLR4 has a primary role in asbestos sensing by macrophages is so far unknown.

In the present work, to shed light on the influence of fiber dimensions in steering macrophage activation and response to amphibole asbestos, cultured murine J774 cells and peritoneal macrophages were exposed to asbestos fibers having different physicochemical features, including size and surface oxidation state. A set of two amosite fibers, short (SFA) and long (LFA), already employed in a large variety of in vitro and in vivo analysis [30,31,32,33], was used. We demonstrate that exposure to SFA activates TLR4 and induces IL-1α release after caspase-1-regulated pyroptosis, an immunogenic lytic cell death subtype [34]. In contrast, LFA induces pro-apoptotic caspase activation and TNF-α extracellular release via ROS, scavenger receptors, and TLR4. This study provides new insights into the mechanism regulating asbestos toxicity, which may be extended to other asbestiform fibrous minerals potentially harmful for human health.

## 2. Results

### 2.1. Short Fibers of Amosite (SFA) Induce Cytotoxicity, Membranolysis and IL-1α Release, While Long Fibers of Amosite (LFA) Only Trigger TNF-α Release

**Figure 1:**
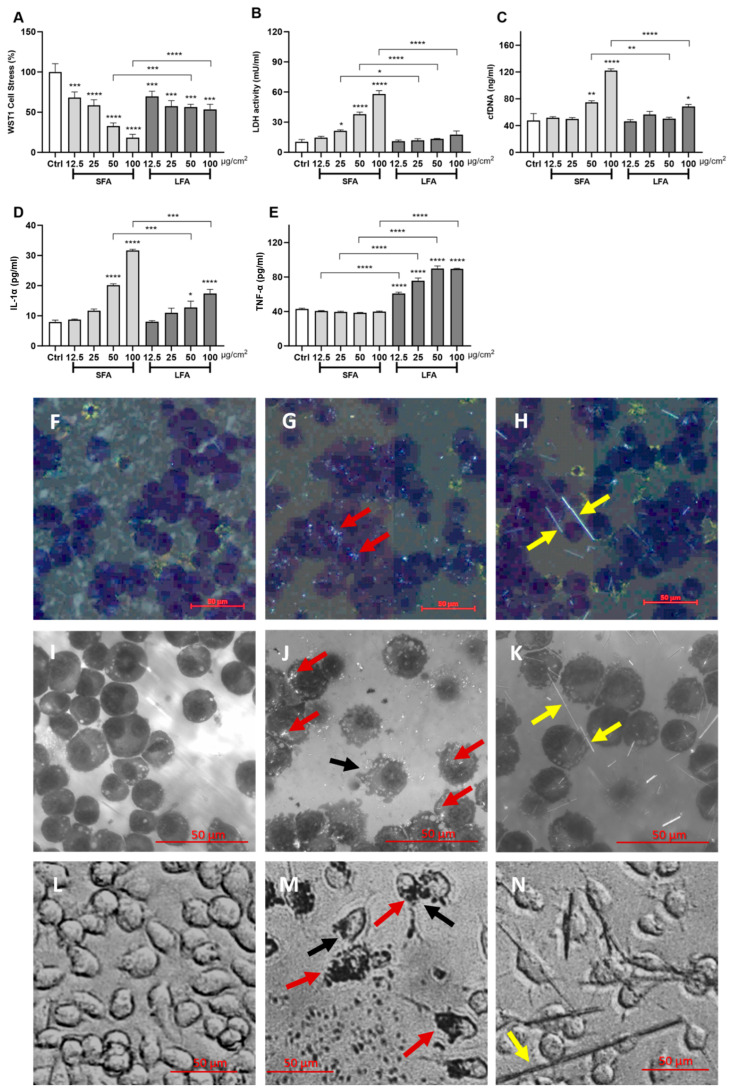
SFA and LFA are differently cytotoxic and pro-inflammatory in vitro. J774 macrophages were exposed 24 h to increasing concentrations (0, i.e., untreated or CTL–12.5–25–50–100 µg/cm^2^) of SFA and LFA. (**A**) mitochondrial stress; (**B**) extracellular LDH activity; (**C**) cell-free DNA extracellular content; (**D**) IL-1α extracellular secretion; (**E**) TNF-α extracellular release. SFA and LFA-exposed macrophages (3 h, 12.5 µg/cm^2^) were deposed on cytospin slides and stained with diff-quick. Polarized light images were collected using a slide scanner (**F**): Ctrl, (**G**): SFA-exposed macrophages, (**H**): LFA-exposed macrophages) or via confocal microscopy (**I**): Ctrl, (**J**): SFA-exposed macrophages, (**K**): LFA-exposed macrophages). Images of macrophages exposed for 3 h to SFA or LFA (12.5 µg/cm^2^) collected in transmitted light (**L**): Ctrl, (**M**): SFA-exposed macrophages, (**N**): LFA-exposed macrophages). Red arrows: SFA internalized by macrophages. Yellow arrows: LFA interacting with macrophages via external contact. Black arrows: dying cells. Determinations were performed in quadruplicate and expressed as the mean ± standard deviation (SD). Data from one representative experiment out of three, which show the same trends, are depicted. Differences between the control cells not exposed to fibers (Ctrl), amosite-exposed cells, and LFA-SFA-exposed cells were evaluated with a one-way ANOVA and Tukey’s multiple comparison test. * *p* < 0.0332, ** *p* < 0.0021, *** *p* < 0.0002, and **** *p* < 0.0001 vs. control, or SFA vs. LFA.

Reliable assays predicting the overall in vivo inflammogenic and fibrotic activities of asbestos exist and include fiber-exposed macrophage cell lines. To compare the in vitro reactivity of SFA and LFA (75th percentile of the cumulative size distribution at ca. 1.5 µm and 9 µm, respectively), we exposed J774 mouse macrophages to increasing doses (12.5, 25, 50, and 100 µg/cm^2^, 24 h) of amosite fibers. Cytotoxic activity was investigated by determining mitochondrial stress (WST-1 assay) and plasma membrane lysis (extracellular LDH and cell-free DNA—cfDNA—levels). SFA induced a larger cytotoxic response with respect to LFA. Mitochondrial activity was significantly impacted by both SFA and LFA in a dose-response fashion. This outcome was, however, stronger with SFA than LFA, in particular at 50 and 100 µg/cm^2^ (Figure 1A). SFA treatment also resulted in significantly more membranolysis (extracellular LDH and cfDNA) than LFA (Figure 1B,C). The pro-inflammatory potential was evaluated by quantifying extracellular IL-1α and TNF-α content. SFA exposure triggered larger dose-dependent IL-1α secretion than LFA, especially at 50 and 100 µg/cm^2^ (Figure 1D). On the contrary, LFA led to a strong dose-dependent release of TNF-α in the extracellular medium, while no increase with respect to the control cells was detected for SFA (Figure 1E). These results indicated the presence of two distinct response mechanisms.

Representative images of cytospin slides of J774 macrophages exposed for 3 h to short and long fibers (12.5 µg/cm^2^) from polarized light microscopy (PLM) showed that SFA were largely internalized (Figure 1G, red arrows). In contrast, LFA was not internalized but interacted with cells via external contact (Figure 1H, yellow arrows). Confocal PLM (Figure 1I: control cells, Figure 1J: SFA, Figure 1K: LFA) on the same slides supported the internalization of SFA (Figure 1J, red arrows) and the interaction of LFA with the exoplasmic leaflet face of macrophages (Figure 1K, yellow arrows). Also, confocal and inverted microscopy (Figure 1L: control cells, Figure 1M: SFA, Figure 1N: LFA) highlighted the significant difference in size distribution between SFA (red arrows) and LFA (yellow arrows) and showed the presence of dying cells characterized by membrane blebs following SFA exposure (black arrows, Figure 1J,M). Overall, our in vitro data demonstrate that SFA is rapidly taken up by macrophages and induce lytic cell death. Conversely, LFA is poorly internalized and, although it activates macrophages, is less cytotoxic when compared to SFA.

### 2.2. SFA Induces Casp-1 Activation and Pyroptotic Cell Death, While Proapoptotic Caspases Are Activated following LFA Treatment

**Figure 2:**
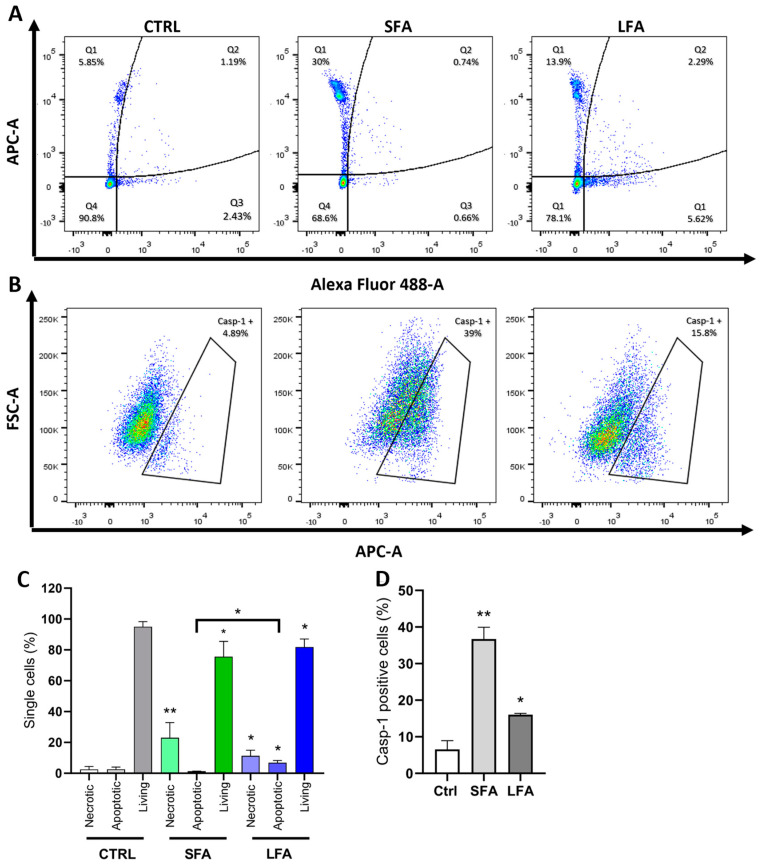
SFA treatment induces casp-1 activation and pyroptosis, while LFA triggers casp-3 and -7 activation and apoptosis. (**A**) Representative sample of flow cytometric analysis of J774 macrophages treated with the CellEvent Caspase-3/7 Green Flow Cytometric Assay kit (Thermo Fisher Scientific, Waltham, MA, USA) to detect casp-3 and casp-7 activity (Alexa Fluor 488-A) and necrotic cells (APC-A) after 24-h exposure to SFA or LFA (25 µg/cm^2^); Q1 (pure necrosis): inactive casp-3/-7; Q2 (late apoptosis) and Q3 (early apoptosis): active casp-3/-7; Q4: healthy cells. (**B**) Representative sample of flow cytometric analysis of J774 macrophages treated with the FLICA 660-Caspase-1 assay kit (ImmunoChemistry Technologies, Davis, CA, USA) to detect casp-1 activity (APC-A) after 16 h exposure to SFA or LFA (25 µg/cm^2^). (**C**) Graphical representation of the percentage of cells in each quadrant as described in (**A**). (**D**) Graphical representation of the casp-1 positive cells as described in (**B**). Results represent a biological replicate carried out in triplicate and are expressed as the mean ± SD. Differences between the control unexposed to particles (Ctrl) and SFA- or LFA-exposed cells, or SFA-apoptotic vs. LFA-apoptotic, were evaluated with one-way ANOVA and Tukey’s multiple comparison test. * *p* < 0.0332, ** *p* < 0.0021 vs. respective control cells.

To better detail the mechanism orchestrating the response to differently-sized asbestos fibers, we investigated casp-3, casp-7, and casp-1 activation in macrophages exposed to SFA or LFA by flow cytometry. First, we observed that SFA treatment induced significant necrotic cell death (Q1: Alexa Fluor negative, APC-A positive) when compared to non-exposed cells without activating pro-apoptotic casp-3 or casp-7 (Figure 2A,C). Exposure to LFA resulted in necrotic cell death, although to a lesser extent than SFA (30% vs. 13.9%) (Figure 2A). Significant casp-1 activation was observed in SFA-treated cells (39% of single cells), while fewer casp-1 positive cells (15.8%) were detected upon treatment with LFA (Figure 2B,D). Contrary to SFA, an increase in casp-3 and casp-7 positive cells was noticed (Q2-late apoptotic: APC-A and Alexa Fluor double-positive, and Q3-early apoptotic: APC-A negative, Alexa Fluor positive), suggesting the onset of apoptotic cell death after LFA exposure (LFA 8% of apoptotic cells vs. 1.4% for SFA) (Figure 2A,C). These results indicate that the phagocytosis of SFA leads to pyroptotic cell death following casp-1 activation. On the other hand, LFA exposure is followed by apoptotic cell death. This suggests a specific response to LFA driven by an additional ICD-related mechanism other than exclusive inflammasome activation, which appears specific to internalizable SFA.

### 2.3. TLR4 Modulates Macrophage Activation and IL-1α upon SFA While MARCO, ROS and TLR4 Trigger LFA-Related Macrophage Responses

**Figure 3:**
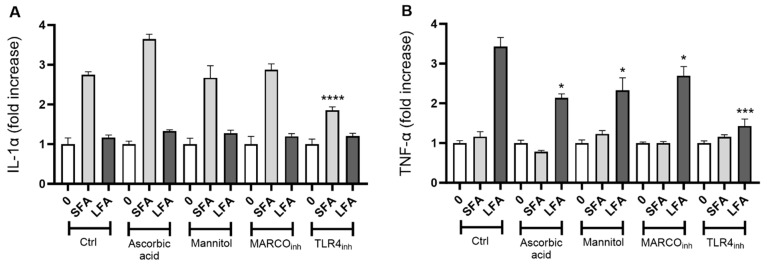
Macrophage activation in response to SFA is initiated by Toll-like receptor 4 (TLR4), while LFA also implicates scavenger receptor MARCO sensing and the ROS cascade. Extracellular IL-1α secretion (**A**) or TNF-α release (**B**) by J774 macrophages incubated 24 h with SFA or LFA (100 µg/cm^2^) alone or by pretreating with ROS scavengers (ascorbic acid, 0.68 nM; mannitol, 50 mM) or innate receptor inhibitors (MARCO_inh_, 0.1 µg/mL; TLR4_inh_, 5 µM). Data from one representative experiment out of three, showing the same trends, are depicted. Determinations were performed in quadruplicate and expressed as the mean ± SD. Differences between groups treated with fibers only and groups treated with fibers + inhibitors were evaluated via one-way ANOVA, followed by Tukey’s multiple comparison test. * *p* < 0.0332, *** *p* < 0.0002, and **** *p* < 0.0001 vs. control.

We next explored the mechanism leading to macrophage activation by targeting the reactive oxygen species (ROS) cascade, the scavenger receptor MARCO (SR-A6), or Toll-like receptor 4 (TLR4). The secretion of IL-1α by exposed macrophages to SFA (24 h, 100 µg/cm^2^) was not modulated by ascorbic acid or mannitol treatment (Figure 3A). TLR4 inhibition, but not MARCO, strongly impacted IL-1α secretion. Consistently with previous data, LFA exposure induced poor IL-1α secretion, which was not affected by the different treatments (Figure 3A). LFA-induced TNF-α release was not only prevented after TLR4 targeting but also by MARCO or ROS cascade inhibitors (Figure 3B). These results indicate that the activation mechanism of macrophages is tightly related not only to the size of the fibers, but also to surface reactivity. Indeed, we noticed a specific TLR4-driven sensing of SFA leading to IL-1α secretion, and a multi-trigger-driven mechanism implicating MARCO, ROS, and TLR4 for TNF-α release upon LFA exposure.

### 2.4. SFA-Related Pyroptotic Response Is Modulated by GSDMD Pores

**Figure 4:**
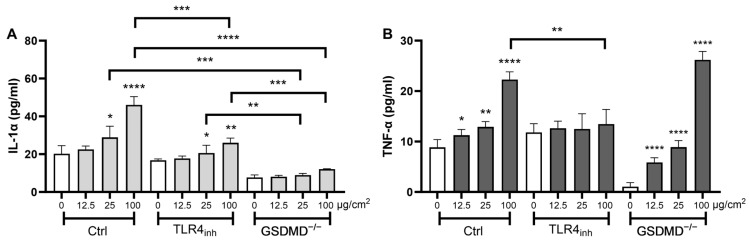
GSDMD pores regulate SFA-induced pro-inflammatory activity in vitro. Extracellular IL-1α secretion (**A**) or TNF-α release (**B**) from wild-type macrophages (Ctrl), Ctrl macrophages pretreated with the TLR4 inhibitor TAK-242 (TLR4_inh_, 5 µM), or GSDMD-deficient (GSDMD^−/−^) peritoneal macrophages incubated 24 h with increasing concentrations (12.5, 25, and 100 µg/cm^2^) of SFA (panel A) or LFA (panel B). Data from one representative experiment out of three, showing the same trends, are depicted. Determinations were performed in quadruplicate and expressed as the mean ± SD. Differences between groups unexposed vs. exposed to fibers and Ctrl vs. TLR4_inh_ or Ctrl vs. GSDMD^−/−^ were evaluated via one-way ANOVA, followed by Tukey’s multiple comparison test. * *p* < 0.0332, ** *p* < 0.0021, *** *p* < 0.0002, and **** *p* < 0.0001 vs. respective control cells.

To clarify the mechanism defining the pyroptotic-related response of macrophages to SFA, we exposed primary peritoneal macrophages obtained from wild-type (Ctrl) and GSDMD-deficient (GSDMD^−/−^) mice. GSDMD is a key pore protein playing a crucial role in pyroptotic ICD [35], and actively involved in IL-1α secretion from macrophages after silica treatment [36,37]. In line with our observations in J774 macrophages, SFA induced sustained dose-dependent IL-1α release in primary macrophages, which was quenched after TLR4 targeting (Figure 4A). This confirmed the key role of TLR4 in driving the macrophage response to SFA. In GSDMD^−/−^ macrophages, SFA-induced IL-1α secretion was dramatically lower, especially at the highest doses (25 and 100 µg/cm^2^), when compared to macrophages in standard conditions (Ctrl) or treated with the selective inhibitor of TLR4 (TLR4_inh_). In contrast, GSDMD deficiency did not affect TNF-α release in LFA-exposed macrophages. Overall, these observations demonstrate that GSDMD pores are crucial in modulating the response to SFA, and further confirm the importance of pyroptosis and TLR4 in macrophage responses to short asbestos. Also, our findings indicate that this pathway is not operative during the response to LFA.

## 3. Discussion

**Figure 5:**
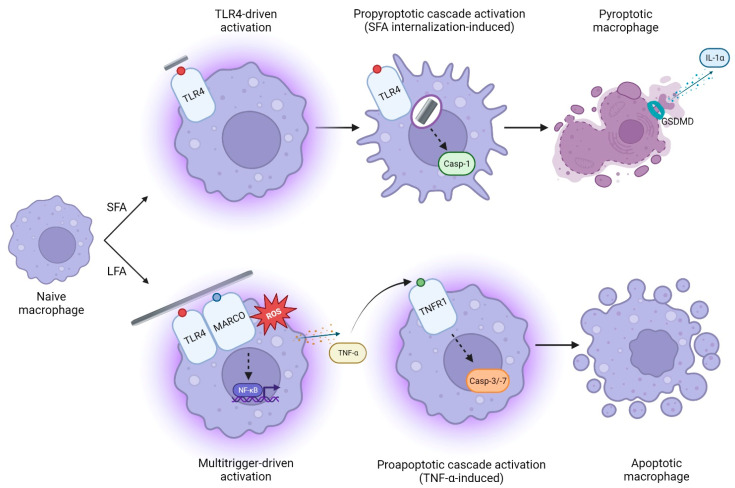
Short (SFA) and long (LFA) amosite asbestos fibers distinctly trigger macrophage activation via different axis, resulting in specific cytokine release (IL-1α vs. TNF-α, respectively) and cell death pathways (pyroptosis vs. apoptosis, respectively). TLR4 guides the response to SFA, which leads to casp-1 activation, GSDMD pore formation, IL-1α secretion, and immunogenic pyroptosis after internalization. In contrast, the response to Fe^2+^-rich, non-internalizable LFA is orchestrated not only by TLR4 but also by MARCO and ROS, and specifically results in apoptosis and TNF-α release. We hypothesize that, through TNFR1-driven interaction, TNF-α can induce apoptosis in cells not experiencing direct contact with long fibers.

In this study, we demonstrated that exposure to short (SFA) or long (LFA) amosite asbestos fibers triggers different activation mechanisms, inducing distinct pro-inflammatory responses in macrophages. Indeed, we found that the mechanism following LFA exposure was mediated by ROS, MARCO, and TLR4, which were necessary for full macrophage activation. TLR4, but not MARCO or ROS, was found to be crucial in SFA response. We demonstrated that SFA exposure dramatically induced cell stress, casp-1 activation, and GSDMD pore assembly, resulting in IL-1α secretion and pyroptotic ICD. The response to LFA was less strong for cell metabolism but included TNF-α release and activation of the pro-apoptotic caspases-3/-7. These data unveil, for the first time, distinct inflammatory mechanisms linked to the dimensions of the fibers, eventually resulting in different outcomes (Figure 5). Our results were in their own way surprising, since several investigations support greater toxicity of LFA than SFA, in particular when incubated with epithelial cells [30,32]. However, a seminal study carried out in a context similar to ours showed that, depending on the dose or the investigated outcome (i.e., TNF-α release), the biological reactivity among SFA and LFA was comparable in murine macrophages [31]. This suggests that, when assessing the cytotoxic potential of possible toxicants, the in vitro model chosen plays a major role.

SFA, in particular, after being sensed by TLR4, was rapidly internalized by exposed macrophages. This process triggered cell stress and induced the NLRP3 inflammasome, as indicated by casp-1 activation. Activated casp-1 recognized and cleaved GSDMD, allowing the formation of GSDMD pores in the plasma membrane, resulting first in the active secretion of IL-1α and then lytic cell death via pro-inflammatory pyroptosis by perturbing the intracellular regulation of ions and water [38,39]. IL-1α is implicated in the expression of pro-inflammatory cytokines and chemokines, resulting in the accumulation of immune cells (monocytes and neutrophils) at the injured site [40]. Exposure to inorganic particles, including crystalline silica, induced the in vitro and in vivo secretion of IL-1α, which plays a major role in silica-induced inflammation [37,41]. Our observation is in line with literature data showing IL-1α transcript upregulation and protein release from human primary airway epithelial cells (HAECs) and primary macrophages exposed to short fibers of amosite and crocidolite asbestos (average length < 5 µm) [42,43]. Also, crocidolite fibers ranging from a few hundred nanometers to several dozen micrometers were found to induce casp-1 activation, which plays a major role in IL-1α maturation and secretion [27,36,44]. 

The above-described mechanism likely occurred for the fraction of LFA whose size allowed them to be engulfed by macrophages. This fraction may include small fibers (L < 5 µm) and cleavage fragments (probably also characterizing SFA) generated upon mechanical stress [45]. These objects, when showing fine/ultrafine size, are hardly detected by usual optical microscopy [46,47]. Despite the uncertainty regarding the pathogenic impact of such particles because of insufficient evidence, it was shown that fragments shorter than 200 nm contribute to lung cancer development [45,48]. Internalizable small fibers and cleavage fragments may be responsible for minor casp-1 activation and IL-1α release observed in LFA-exposed macrophages, further supporting our main finding.

TLR4 is a member of the pattern recognition receptors (PRRs) family, known to be involved in gram-negative lipopolysaccharide (LPS) and DAMPs sensing [49,50]. The implications of TLR4 in chronic inflammation and mesothelioma development following asbestos inhalation are hypothesized [51]. However, the direct role of TLR4 in regulating macrophage activation following asbestos exposure is so far undemonstrated. In line with our observations, it was noticed that TLR4 contributed to the in vitro and in vivo pro-inflammatory activity of carbon nanotubes, which share some morphological features with asbestos, including fibrous needle-like shape, biopersistence, and the ability to adsorb biochemical macromolecules [29,52]. In addition, some studies have demonstrated the involvement of TRL4 in the cellular pro-inflammatory response to ambient particulate matter and crystalline silica [26,28]. These findings and our data showing that both SFA and LFA induce macrophage activation via TLR4 strongly suggest that TLR4 is crucial for innate immune recognition, and for orchestrating the inflammatory response to fibers with diverse size and physicochemical properties.

Our data support the role of MARCO as a signaling receptor for long asbestos fibers. MARCO, a class A scavenger receptor, mediates innate recognition and clearance of pathogens and environmental particles [53,54]. Inorganic fibers such as carbon nanotubes were found to be internalized via a MARCO-orchestrated pathway in epithelial cells [55]. One study demonstrates that human alveolar macrophages collected from patients with asbestosis overexpress MARCO, indicating that this scavenger receptor may contribute to asbestos-induced lung fibrosis [23]. Similarly to our results, the same study suggests that MARCO mediates the toxic impact of long-chrysotile asbestos on murine alveolar macrophages. Furthermore, it also reveals the sustained generation of ROS following asbestos exposure, which is a well-recognized process for long fibers [56]. In line, we observed that the response to LFA (and not SFA) was largely impacted by the modulation of the ROS cascade. This agrees with SFA and LFA chemical characteristics, which indicate a higher surface oxidation state for ultrasonicated SFA in comparison to LFA (table in Section 4.1). Similarly, previous observations also demonstrated different potentials in free radical generation between non-sonicated SFA and LFA [30]. As observed in our study, a large content of poorly coordinated Fe^2+^ ions on the surface of LFA was detected [30]. Such Fe^2+^ cations, still detectable to a large extent on the surface of ultrasonicated LFA (table in Section 4.1), can promote sustained oxidative stress, resulting in pro-inflammatory activity [57]. It is also known that signaling pathways activating NF-kB and consequent TNF-α expression can be modulated by ROS, proposing a mutual positive influence between ROS generation and TNF-α upregulation [58]. These concepts explain our observation regarding the impact of ascorbic acid or mannitol only on LFA-exposed cells and point out that, in addition to morphological characteristics, chemical features (i.e., surface oxidation state) play a role in driving the different in vitro responses to the fibers.

LFA exposure also led to apoptosis orchestrated by casp-3 and -7, commonly activated during apoptotic cell death [59]. This is consistent with previous results observed with different asbestos types, including chrysotile and Libby amphibole asbestos [15,60,61]. It is known that TNF-α, a powerful regulator of pro-inflammatory cytokine expression in response to Toll-like receptor activation [62], triggers apoptosis or apoptosis-driven secondary necrosis (late apoptosis) via TNF receptor 1 (TNFR1) [63,64]. Therefore, TNF-α released by macrophages in response to long fibers may be recognized by a fraction of fiber-free cells and lead to “indirect” apoptosis following TNFR1, caspase-3, and caspase-7 activation. In this context, it was noticed that TNF-α release from alveolar macrophages in patients with asbestosis, likely exposed to long fibers, was pivotal in the pathogenesis of the disease [65].

Our observations imply that SFA is not biologically inert, since SFA-induced pyroptotic cell death can activate an immunogenic response and stimulate local inflammation. The general low pulmonary pathogenic potential of short fibers mentioned in the literature may be due to clearance or fiber detoxification processes [66]. Indeed, short fibers can be removed from the lungs by several mechanisms, including transport through the tracheobronchial ciliated mucous or the lymphatic system [10]. This matter, which reflects a common limitation of in vitro assessments, will be deeply investigated in a forthcoming in vivo study.

## 4. Methods and Materials

### 4.1. Short (SFA) and Long (LFA) Fibers of Amosite Asbestos

Samples of short (SFA) and long (LFA) fibers of amosite asbestos were kindly supplied by Dr. Lang Tran from the Institute of Occupational Medicine (Edinburgh, UK). These samples were originally prepared from the same batch of South African amosite at Johns Manville Corporation, Littleton (Denver, CO, USA). Short fibers were obtained by grinding LFA in a ceramic ball mill machine (agate mortar), followed by water sedimentation [67]. A total of 37% of the resulting material was described as fibers, in accordance with the 1986 WHO definition [67,68].

**Figure 6:**
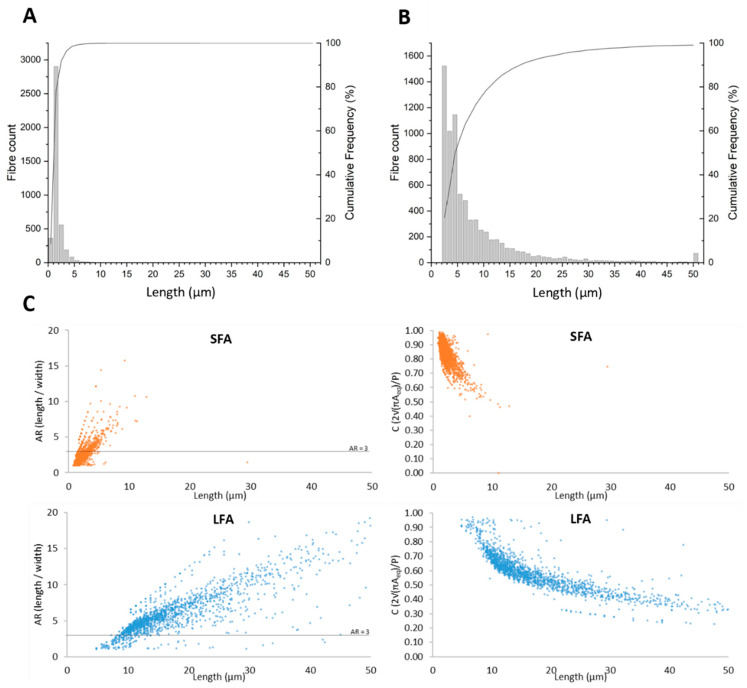
Length frequency and cumulative distribution plots (bars and curves, respectively) of SFA (**A**) and LFA (**B**). (**C**) Length vs. Aspect Ratio (AR) and Length vs. Circularity of SFA and LFA. FPIA data evidence large-dimensional and morphometric differences between SFA and LFA. Dimensional characterization shows that a significant amount of SFA and LFA have elongated morphology (AR ≥ 3) and low circularity, indicating that the partial fibrosity of SFA is maintained after ultrasonication.

Before incubation with the cells, SFA and LFA stock suspensions (3.2 mg/mL, Vol = 3 mL) in 1X phosphate buffered saline (PBS) were ultrasonicated on ice (100 W: 45 min; probe diameter 3 mm) using a VCX 750 ultrasonic processor (Sonics, Newtown, CT, USA). This procedure dissociates fiber bundles and allows their better suspension and diffusion in cell culture medium. Size distributions of SFA and LFA suspensions following sonication were obtained by automated flow particle image analysis using the Sysmex FPIA-3000 apparatus (Malvern Instruments, Malvern, UK). Each sample was run four times with an objective lens at 20× magnification in both High- and Low-Power field modes. Statistical analysis was then performed on the four runs to obtain SFA and LFA size distributions. Both SFA and LFA contain a significant number of elongated particles, defined as objects having Aspect Ratio (AR) Length/Width ≥ 3 [69]. For Fe speciation, ultrasonicated SFA and LFA (0.5 mg/mL) were incubated in 1 mM ferrozine (to evaluate the amount of removable Fe^2+^) or 1 mM ferrozine and 1 mM ascorbic acid (to evaluate the total amount of removable iron Fe_tot_ = Fe^2+^ + Fe^3+^). The kinetics of leached Fe^3+^ were obtained by subtracting Fe^2+^ from Fe_tot_. The amount of iron extracted after 1, 5, 24, and 48 h was quantified spectrophotometrically (Unikon 930, Kontron Instrument, Augsburg, Germany) in the supernatant by measuring the absorption of the Fe^2+^-ferrozine complex at 562 nm (ε_mM_ = 27.9 mM^−1^cm^−1^). SFA and LFA properties are summarized in Figure 6 and Table 1.

### 4.2. Cell Culture and In Vitro Exposure

The J774 mouse macrophage cell line (ATCC#TIB-67) was grown to pre-confluence (70% confluence) in Dulbecco’s modified Eagle Medium (DMEM GlutaMAX) supplemented with 10% fetal bovine serum (FBS) and 1% of antibiotic-antimycotic (AA) (fungizone (25 μg/mL) and penicillin-streptomycin (100 U/mL and 100 μg/mL). Before fiber exposure, cells were seeded in 96-well plates (50,000 cells/well) and allowed to adhere for 4 h at 37 °C, 5% CO_2_. Fibers were heat-sterilized at 200 °C for 2 h just prior to the exposure to Inactivate any trace of endotoxin, and stock suspensions of SFA or LFA in DMEM GlutaMAX (3.2 mg/mL, Vol = 3 mL) were prepared via ultrasonication as described above. SFA and LFA suspensions or serum-free DMEM GlutaMAX (negative control) were distributed in four replicates in the plates to the final concentrations of 12.5–25–50–100 μg/cm^2^, and incubated for 24 h at 37 °C in a 5% CO_2_ atmosphere. Optical imaging of cells exposed to SFA or LFA (3 h, 12.5 μg/cm^2^) was carried out in transmitted light using a Leitz Labovert inverted microscope (Leitz, Oberkochen, Germany).

The role of the ROS cascade was Investigated by using two radical scavengers: mannitol, a hydroxyl radical (^•^OH) scavenger [71], and ascorbic acid, one of the most potent non-enzymatic antioxidants [72]. In the experiments with inhibitors, fiber exposure was carried out by treating the cells with anti-MARCO antibodies (clone ED31, product code MCA1849T, 0.1 µg/mL) purchased by Bio-Rad (Hercules, CA, USA) to inhibit MARCO receptors [73], ascorbic acid (0.68 mM), one of the most potent non-enzymatic antioxidants [72], mannitol (50 mM), a hydroxyl radical (^•^OH) scavenger [71], and TAK-242 (5 µM) used to inhibit TLR4 [28]. All reagents were purchased from Sigma Aldrich (St. Louis, MO, USA), unless stated otherwise.

### 4.3. Peritoneal Macrophage Culture

Primary peritoneal macrophages were collected from C57BL/6 mice and GSDMD-deficient mice following a procedure established by Arras [74,75,76]. After mice were sacrificed, the peritoneum was lavaged with 7 mL of sterile 0.9% NaCl. The cells were then washed, counted, and dispersed in DMEM GlutaMAX supplemented with 10% fetal bovine serum (FBS) and 1% antibiotic-antimycotic. Cells were then plated in 96-well culture plates at a density of 10^6^ macrophages/mL. Cultures were incubated for 18 h at 37 °C and 5% CO_2_ in a humidified incubator. On the second day of culture, the cells were washed with 1 mL of PBS (2 × 500 µL) to remove nonadherent cells and then exposed for 24 h to serum-free DMEM GlutaMAX containing different concentrations of the tested compounds (LFA or SFA, 12.5–25–100 μg/cm^2^) in four replicates. Control cells were exposed to serum-free DMEM GlutaMAX, not containing fibers, in four replicates.

### 4.4. In Vitro Cytotoxicity Assessment 

The cytotoxic impact of the fibers on J774 macrophages was investigated after 24 h exposure by measuring the mitochondrial metabolic activity via WST-1 assay [77] and by evaluating cell membrane integrity via lactate dehydrogenase (LDH) release assay using an LDH-Glo Cytotoxicity Assay kit (Promega, Madison, WI, USA) [78]. The amount of DNA leakage (cell-free DNA, cfDNA) in the cell culture supernatant following fiber-induced cell death [79] was assessed using a Quant-iT PicoGreen dsDNA reagent (Invitrogen, Carlsbad, CA, USA), according to the manufacturers’ protocols. Absorbance at 440 nm (WST-1), luminescence (LDH), and fluorescence (cfDNA) were monitored using a SpectraMax iD3 microplate reader (Molecular Devices, San Jose, CA, USA).

### 4.5. Determination of Cytokines (IL-1α, TNF-α)

The pro-inflammatory potential of the fibers was evaluated in vitro by quantifying the amount of IL-1α and TNF-α released in the cell culture supernatant collected after fiber exposure using DuoSet ELISA kits (IL-1α: DY400, detection limit 5 pg/mL; TNF-α: DY410, detection limit 31.2 pg/mL; R&D Systems, Minneapolis, MN, USA) according to the manufacturer’s guidelines. Absorbance was determined using a SpectraMax iD3 microplate reader (Molecular Devices, San Jose, CA, USA) set to 450 nm, with wavelength correction set to 540 nm.

### 4.6. Cytological Analysis

Cytospin is a cytological technique that allows for the investigation of cell populations upon staining by optical microscopy [80]. To this end, J774 macrophages were exposed for 3 h to 12.5 µg/cm^2^ of fibers. Then, cells were harvested by gentle scraping and centrifuged (166.63× *g*) to deposit a cell layer on glass slides. Cells were fixed with ice-cold methanol (5 min) and stained via Diff-Quick staining according to the manufacturer’s guidelines (Polysciences Inc., Warrington, PA, USA). Cytospin slides were examined in polarized light microscopy (PLM) with a Zeiss LSM800 confocal microscope and a Zeiss Axioscan slide scanner (Carl Zeiss, Oberkochen, Germany).

### 4.7. Flow Cytometric Analysis of Caspases-3/-7 and Caspase-1 Activity

Caspase-3 (casp-3) and caspase-7 (casp-7) activation was determined by using the CellEvent™ Caspase-3/7 Green Flow Cytometry Assay Kit (Thermo Fisher Scientific, Waltham, MA, USA). The membrane-permeable substrate is cleaved by activated caspase-3 or caspase-7 in apoptotic cells, allowing the dye to bind to DNA and inducing a green fluorescence signal [59]. Caspase-1 activation as a marker of necrotic (pyroptotic) cell death was evaluated using the FLICA 660-Caspase-1 assay kit (ImmunoChemistry Technologies, Davis, CA, USA) substrate [81]. The FLICA reagent 660-YVAD-FMK enters cells and irreversibly binds to activated caspase-1, generating a far-red fluorescent signal. Unbound reagent diffuses out of cells and is washed away.

J774 macrophages were seeded on transparent 24-well plates (200,000 cells/well) and then exposed for 24 h (caspase-3 and caspase-7) or 16 h (caspase-1) in triplicate to a fiber concentration of 25 μg/cm^2^. After trypsinization and washing with PBS (3 × 500 µL), cells were collected in FACS tubes. For caspase-3/-7 analysis, cells were incubated with 200 µL of a 500 nM caspase-3/7 green detection reagent solution in PBS for 30 min at 37 °C. During the last 5 min, 1 µL of a 1 mM SYTOX AADvanced dead cell stain solution was added to the tubes to stain dead cells. For caspase-1 analysis, harvested cells were incubated with FLICA 660 reagent diluted 1:45 in PBS (200 µL/tube) for 1 h at 37 °C. Then, cells were washed and resuspended in PBS. 

Quantitative analysis of 10,000 cells per sample was performed on a FACS Canto II flow cytometer (BD Biosciences, Franklin Lakes, NJ, USA) and analyzed using FlowJo V10 software (*n* = 3 independent experiments).

## 5. Conclusions

It is well accepted that only longer fibers explain asbestos-related detrimental effects. Nevertheless, it is crucial to underscore that the toxicity of short fibers should not be underestimated or disregarded. Our study provides novel insights on the molecular mechanisms orchestrating the response of macrophages to short and long amosite fibers. The accumulated evidence highlights the key role of TLR4, a receptor known to be involved in the pro-inflammatory response to inorganic respirable objects. Also, our results confirm the importance of MARCO and ROS in modulating the response to long fibers, characterized by the release of TNF-α. In contrast, GSDMD and inflammasome activation orchestrate an IL-1α-related pro-inflammatory response to cell-internalizable short fibers. Our novel paradigm and pathways should be considered when establishing predictive toxicology models and issuing warnings regarding the potential inflammatory and toxic activity of short asbestos fibers.

## Figures and Tables

**Table 1:** Main physicochemical properties of SFA and LFA.

		SFA	LFA
Particle size distribution ^+^ (quartiles: 25/50/75%) (μm)	P_25_	0.5	3.0
	P_50_	1.0	5.0
	P_75_	1.5	9.0
Specific surface area * (m^2^/g)		11	6
Surface Fe/Si ° (at. %)		0.91 ± 0.03	0.89 ± 0.03
Average surface oxidation state ^#^		SFA > LFA	
Surface oxidation state—Binding energy ^$^ (eV)		56.6	55.8
Leachable Fe at 24 h (Fe^3+^ + Fe^2+^, ng Fe/mg amosite) ^§^		Fe_tot_ = 20.4 ± 0.8	Fe_tot_ = 13.6 ± 1.3
		Fe^3+^ = 16.6 ± 0.6	Fe^3+^ = 7 ± 1.2
		Fe^2+^ = 3.8 ± 0.2	Fe^2+^ = 6.6 ± 0.2
Particle-derived free radicals ^£^		Moderate	Very high

^+^ Measured by a flow particle image analyzer (FPIA) in PBS. The size distribution of sonicated SFA and LFA used in this study is equivalent to data from [32] obtained on non-sonicated fibers. * N_2_-BET adsorption. Data from [32], obtained on non-sonicated fibers. ° Measured as the ratio between the XPS intensities of the Si2p and Fe3p photolines. Data from [30], obtained on non-sonicated fibers. ^#^ Measured via incubation in ferrozine (1 mM) or ferrozine (1 mM) + ascorbic acid (1 mM). In line with [30], data indicate the presence of iron ions in a higher oxidation state on the SFA surface (Fe^3+^), likely due to the oxidation process that occurred during sample preparation. ^$^ Measured as the XPS Fe3p photopeak position. Data from [30], obtained on non-sonicated fibers. ^§^ Measured after incubation with iron chelators. Data suggest no significant differences in the iron speciation of SFA and LFA following sonication, in line with previous observations on non-sonicated fibers [30,70]. ^£^ Measured as the cleavage of a hydrogen-carbon bond. Data from [30], obtained on non-sonicated fibers. Data indicate that LFA is a more potent radical generator than SFA.

## Data Availability

The dataset used and/or analyzed during the current study is available from the corresponding author under reasonable request.
